# Modulation of the cancer cell transcriptome by culture media formulations and cell density

**DOI:** 10.3892/ijo.2015.2930

**Published:** 2015-03-17

**Authors:** SEUNG WOOK KIM, SUN-JIN KIM, ROBERT R. LANGLEY, ISAIAH J. FIDLER

**Affiliations:** Department of Cancer Biology, Metastasis Research Laboratory, The University of Texas MD Anderson Cancer Center, Houston, TX 77030, USA

**Keywords:** microarray, epithelial-mesenchymal transition, cell culture, media formulation

## Abstract

We investigated how varying the composition of cell culture formulations and growing cancer cells at different densities might affect tumor cell genotype. Specifically, we compared gene expression profiles generated by human MDA-MB-231 breast cancer cells cultured in different media [minimum essential medium (MEM), Dulbecco’s modified Eagle’s medium (DMEM), or Roswell Park Memorial Institute (RPMI)-1640 medium] containing different concentrations of fetal bovine serum (FBS) or different sera (equine or bovine) that were grown at different cell densities. More than 2,000 genes were differentially modulated by at least a 2-fold difference when MDA-MB-231 cancer cells were 90% confluent and compared with cultures that were 50% confluent. Altering the concentration of serum produced an even more pronounced effect on MDA-MB-231 cancer cell gene expression in that 2,981 genes were differentially expressed in a comparison between cells cultured in 0.1% FBS and same cell density cultures that were maintained in 10% FBS. A comparison between MDA-MB-231 cancer cells that were 90% confluent in MEM, DMEM, or RPMI-1640 media, all containing 10% FBS, resulted in 8,925 differentially expressed genes. Moreover, one-quarter (25.6%) of genes from our genome-wide expression analysis were expressed at significantly different levels by cells grown in MEM, DMEM, or RPMI-1640 media. Genes associated with epithelial-mesenchymal transition (EMT) were among the genes that were differentially modulated by cells grown in different cell culture formulations and these genes were verified at the protein level. Collectively, these results underscore the importance of accurate reporting and maintenance of uniform culture conditions to ensure reproducible results.

## Introduction

Recent reports have sought to raise awareness of the growing number of cancer research studies whose findings cannot be independently reproduced (1). Indeed, investigators from the Hematology and Oncology Department at Amgen in Thousand Oaks, CA, USA, were only able to confirm the scientific results in six out of 53 (11%) reports that were regarded as landmark studies (1). A similar assessment of 67 projects (47 of which were oncology studies) by researchers from Bayer HealthCare (Leverkusen, Germany) revealed that only one-quarter of the published data could be reproduced (2). Several reasons have been proposed to explain the high rate of contrasting results among different laboratories, including investigator bias, inappropriate statistical analysis of results, and insufficient sample size (1,2). Investigator bias is a broad category that includes manipulation of the analysis and selective reporting of data (3). It is also well known that the smaller the experimental sample size, the less likely the research findings are to be true (3). Alterations in cell culture conditions are also reported to skew experimental results and increase the likelihood that a study cannot be replicated (4). However, there are no comprehensive analyses of the effects of cell culture modifications on the cancer cell transcriptome.

Cancer cell lines are an indispensable component of a translational research program and have played a critical role in several important discoveries, including identification of *BRAF* mutations in human tumors (5), development of targeted therapeutic agents (6), determining mechanisms of therapeutic resistance (7), and many others (8). The extent that investigators rely on cancer cell lines for their studies is exemplified by the current collection of 200 lung cancer cell lines, which have been the subject of >9,000 citations (9). These and other cancer cell lines are maintained in defined media that are isosmotic and contain a buffer, inorganic salts, nutrients (amino acids and vitamins) and an energy source (usually glucose) to permit normal cell metabolism. However, the composition of media formulations can vary widely. For example, complete Eagle’s minimum essential medium (MEM) contains 1,000 mg/l of glucose, whereas the concentration of glucose in Dulbecco’s modified Eagle’s medium (DMEM) containing the high glucose modification is 4,500 mg/l. The concentration of glucose present in Roswell Park Memorial Institute (RPMI)-1640 medium falls between MEM and DMEM and is 2,000 mg/l.

It is widely known that the tumor microenvironment has a profound impact on determining the gene expression patterns of cancer cells (10). Cancer cells may also influence gene expression of normal (non-transformed) cell populations residing in the tumor microenvironment and the extent of the gene modulation occurring in both compartments may be quantitatively assessed experimentally using cross-species hybridization of microarrays (11). Here, we varied the *in vitro* microenvironment of MDA-MB-231 breast cancer cells by adjusting their cell culture conditions and then constructed gene expression profiles on the cells to determine the possibility that cell culture modifications could contribute to the inability to reproduce experimental results. The resulting data emphasize that in order to obtain reproducible results for cancer cells grown in culture, one must adhere to the precise details regarding media formulation, supplemental nutrition, and the density of the cell preparation at the time of analysis.

## Materials and methods

### Antibodies

The following antibodies were used in this study: anti-IL-8, anti-E-cadherin (Invitrogen Life Technologies, Carlsbad, CA, USA); anti-S100A4, anti-VIM, anti-CD44 (Cell Signaling Technology, Inc., Beverly, MA, USA); anti-CD24 (R&D Systems, Minneapolis, MN, USA); anti-β-actin (AC-15) (Sigma-Aldrich, St. Louis, MO, USA); goat anti-mouse IgG-horseradish peroxidase (HRP), goat anti-rabbit IgG-HRP (Santa Cruz Biotechnology, Inc., Santa Cruz, CA, USA).

### Cell lines and cell culture conditions

Human MDA-MB-231 breast cancer cells (12) were maintained as a monolayer culture in MEM, DMEM, or RPMI-1640 supplemented with L-glutamine, sodium pyruvate, non-essential amino acids, a 2-fold vitamin solution, and penicillin-streptomycin (Invitrogen Life Technologies). Fetal bovine serum (FBS) (HyClone, Logan, UT, USA) or horse serum (HS) (Invitrogen Life Technologies) was added to the media. All tissue culture reagents were free of endotoxin as determined by the Limulus Amebocyte Lysate assay (Associates of Cape Cod, Inc., Woods Hole, MA, USA). MDA-MB-231 cells were free of the following murine pathogens: *Mycoplasma* species, Hanta virus, hepatitis virus, minute virus, adenovirus (MAD1, MAD2), cytomegalovirus, ectromelia virus, lactate dehydrogenase-elevating virus, polyma virus, and Sendai virus (assayed by the Research Animal Diagnostic Laboratory, University of Missouri, Columbia, MO, USA). MDA-MB-231 breast cancer cells were tested at the MD Anderson Characterized Cell Line Core Facility using short tandem repeats DNA profiling.

### Microarray analysis

Total RNA was extracted from the cultured cells by using the mirVana miRNA Isolation kit (Life Technologies, Grand Island, NY, USA) according to the manufacturer’s instructions. The integrity of the RNA fraction was determined using a Bio-Rad Experion Bioanalyzer (Bio-Rad, Hercules, CA, USA) as a surrogate for mRNA quality control. Biotin-labeled cRNA samples were prepared by using the Illumina Total Prep RNA Amplification kit and 1.5 μg of biotinylated cRNA sample was hybridized to HumanHT-12 v4.0 Expression BeadChip (Illumina, Inc., San Diego, CA, USA). BeadChips were scanned with an Illumina BeadArray Reader and the microarray data were normalized using the quantile normalization method in the Linear Models for Microarray Data package in the R language environment (12). All statistical analyses were performed using the BRB-ArrayTools software program (version 4.0) (13).

### Western blot analysis

Western blot analysis was used to confirm the results of the microarray data. MDA-MB-231 cancer cells (2×10^6^ cells) were plated onto 100 mm culture dishes and maintained in the various media formulations containing different concentrations (or types) of sera. Whole-cell lysates of cancer cells were obtained when cancer cells reached the appropriate experimental cell density by lysing cells in buffer [10 mM Tris (pH 8.0), 1 mM EDTA, 0.1% SDS, 1% deoxycholate, 1% NP40, 0.14 M NaCl, 1 μg/ml leupeptin, 1 μg/ml aprotinin, and 1 μg/ml pepstatin] containing a protease inhibitor mixture (Roche Diagnostics, Indianapolis, IN, USA) (12). Next, 30 μg of total protein was separated by electrophoresis on 4–12% Nu-PAGE gels (Life Technologies) and transferred to nitrocellulose membranes. Membranes were blocked for 1 h and then incubated overnight at 4°C with primary antibodies (1:1,000). The membranes were rinsed, incubated with HRP-conjugated secondary antibodies (1:3,000), and visualized by enhanced chemiluminescence (Amersham Pharmacia Biotech, Piscataway, NJ, USA). To ensure equal protein loading, the blots were stripped and reprobed with an anti-β-actin antibody (Sigma-Aldrich). Quantification of protein levels in the western blots was performed using ImageJ software (National Institutes of Health, Bethesda, MD, USA).

### Real-time reverse transcription polymerase chain reaction

Microarray results for interleukin-8 (*IL-8*), *S100A4*, vimentin (*VIM*), E-cadherin (*CDH1*), *CD44*, and *CD24* were validated using real-time reverse transcription polymerase chain reaction (RT-PCR). Total RNA was extracted from the MDA-MB-231 cancer cells using the Qiagen RNeasy Mini kit (Qiagen, Valencia, CA, USA) according to the manufacturer’s instructions. First-strand cDNA was synthesized from 5 μg RNA using SuperScript III Reverse Transcriptase (Invitrogen Life Technologies). RT-PCR was performed using TaqMan^®^ Universal PCR Master Mix and quantified with Applied Biosystems 7500 Real-Time PCR system (Applied Biosystems, Foster City, CA, USA). The following TaqMan^®^ Gene Expression assays were used in our validation study; human *IL-8* (Hs00174103-ml); human *S100A4* (Hs00243202_m1); human *VIM* (Hs00185584_m1); human *CDH1* (Hs01023894_m1); human *CD44* (Hs01075861_m1) and human *CD24* (Hs02379687_s1) (all from Applied Biosystems). 18S rRNA was used as an endogenous control. Relative mRNA expression in the cells was calculated using the ΔΔCt method (14) and the results are expressed as the mean ± standard deviation (SD) of mRNA relative to that of control.

### Statistical analysis

All statistical analyses were performed using BRB-ArrayTools version 4.3.2 under the R language environment. The microarray data were normalized using the quantile normalization method in the Linear Models for Microarray Data package. A two-sample t-test was applied to gene expression data from three groups of samples and expression of genes and a P<0.001 was considered statistically significant. This stringent significance threshold was used to limit the number of false-positive findings. We also performed a global test of whether the expression profiles differed between the classes by permuting the labels of which arrays corresponded to which classes. For each permutation, the P-values were recomputed and the number of genes with significant expression levels of <0.001 was noted. Cluster analyses were performed with the Cluster software program and heat maps were generated using the TreeView software program (15).

### Accession numbers

The microarray data have been deposited in the Gene Expression Omnibus under accession number GSE61670.

## Results

### Cell density affects patterns of gene expression in MDA-MB-231 cancer cells

**Figure 1:**
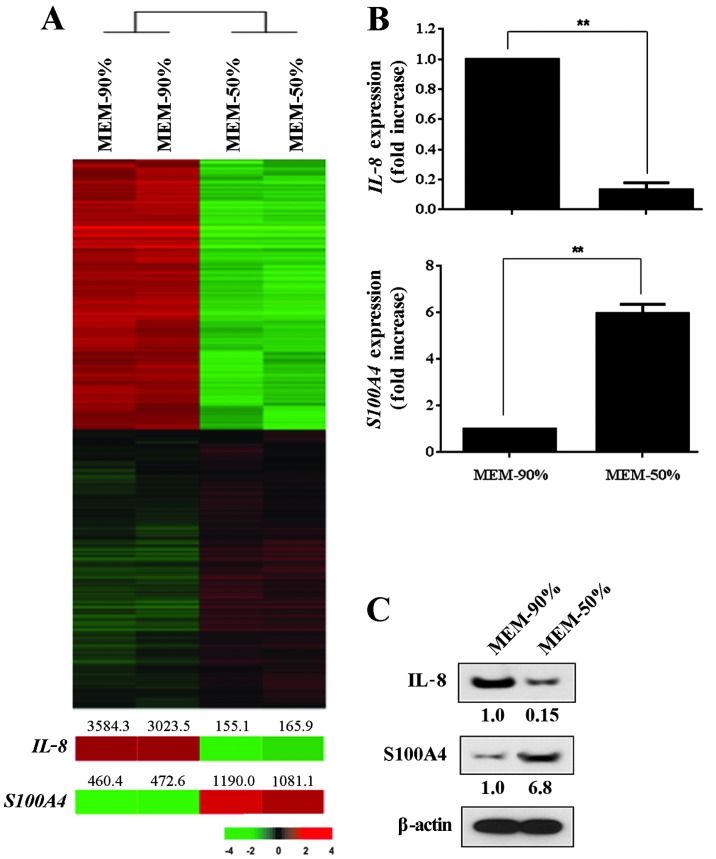
Differential gene expression in confluent (90%) and subconfluent (50%) MDA-MB-231 breast cancer cells. (A) Hierarchical clustering analysis of confluent and subconfluent MDA-MB-231 breast cancer cells. Genes with an expression level exceeding a 2-fold difference relative to the median value were selected for hierarchical clustering analysis (n=2,234 genes). The data are presented in matrix format in which rows represent individual genes and columns represent each culture condition. Each cell in the matrix represents the expression level of a gene feature in an individual culture. The color red or green in cells reflects relative high or low expression levels, respectively, as indicated in the scale bar (log2-transformed scale). (B) Real-time reverse transcription polymerase chain reaction (RT-PCR) validation of interleukin-8 (*IL-8*) and *S100A4* expression using RT-PCR and (C) confirmation of IL-8 and S100A4 protein with western blot analysis. Values shown are means ± standard deviation (SD) from three independent experiments. ^**^P<0.01.

To begin to study the effects of the cell culture environment on the cancer cell transcriptome, we first examined how alterations in cell density affect patterns of gene expression of MDA-MB-231 breast cancer cells. The cells were grown as monolayers in MEM supplemented with 10% FBS and harvested for analysis when they were 50 or 90% confluent in culture dishes. We noted that this alteration in cell density resulted in the differential expression of 2,234 genes (Fig. 1A). Specifically,1,100 genes were significantly upregulated in cancer cells that were 90% confluent when compared to cells that were 50% confluent. A similar number of genes (1,134 genes) were significantly downregulated in 90% confluent cancer cells. We selected *IL-8* and *S100A4* from the upregulated and downregulated gene sets for validation using RT-PCR, because these two genes were among the most differentially expressed in their corresponding gene sets. The expression of *IL-8* mRNA in cells that were 90% confluent in MEM was 9-fold greater than that of cells cultured to 50% confluence in the same medium. In contrast, we noted a 6-fold downregulation in *S100A4* mRNA expression in cells that were 90% confluent in comparison to cells that were analyzed once they reached 50% confluence. We confirmed the differential expression of *IL-8* and *S100A4* at the protein level using western blot analysis (Fig. 1C).

### Concentration of FBS affects MDA-MB-231 cancer cell gene expression

**Figure 2:**
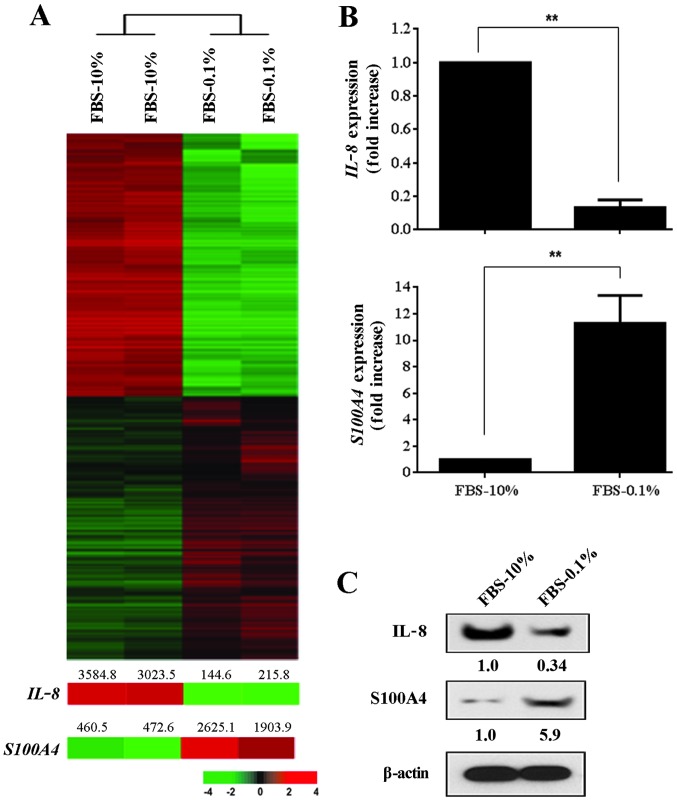
Differential gene expression between MDA-MB-231 breast cancer cells cultured in minimum essential medium (MEM) supplemented with 10 and 0.1% fetal bovine serum (FBS). (A) Hierarchical clustering analysis of cells maintained in MEM containing 10% FBS and MEM containing 0.1% FBS. Genes with an expression level exceeding a 2-fold difference relative to the median value were selected for hierarchical clustering analysis (n=2,981 genes). The data are presented in matrix format as described in Fig. 1. (B) Real-time reverse transcription polymerase chain reaction (RT-PCR) analysis of interleukin-8 (*IL-8*) and *S100A4* expression. (C) Western blot analysis of IL-8 and S100A4. Values shown are means ± standard deviation (SD) from three independent experiments. ^**^P<0.01.

Next, we analyzed how varying the concentration of FBS affected gene expression in MDA-MB-231 breast cancer cells. The concentration of FBS in several same passage cultures of MDA-MB-231 cancer cells was adjusted to either 0.1 or 10% and all cells were analyzed when cultures reached 90% confluency. We found almost 3,000 genes that were differentially expressed between MDA-MB-231 cells cultured in 10% FBS and those cultured in 0.1% FBS (Fig. 2A). A total of 1,489 genes were expressed at significantly higher levels in cells cultured in 10% FBS when compared to cells cultured in 0.1%. Once again, *IL-8* and *S100A4* gene expressions were among the more differentially regulated genes and both were examined in greater detail. RT-PCR analysis revealed that *IL-8* was upregulated by 10-fold in MDA-MB-231 cancer cells that were grown in 10% FBS, whereas *S100A4* was down-regulated by 10-fold in cells maintained in 10% FBS (Fig. 2B). We confirmed these findings by western blot analysis, which showed a 3-fold increase of IL-8 expression in MDA-MB-231 cancer cells grown in 10% FBS and almost a 6-fold increase in S100A4 protein expression when cells are grown in the reduced concentration (0.1%) of FBS (Fig. 2C).

**Figure 3:**
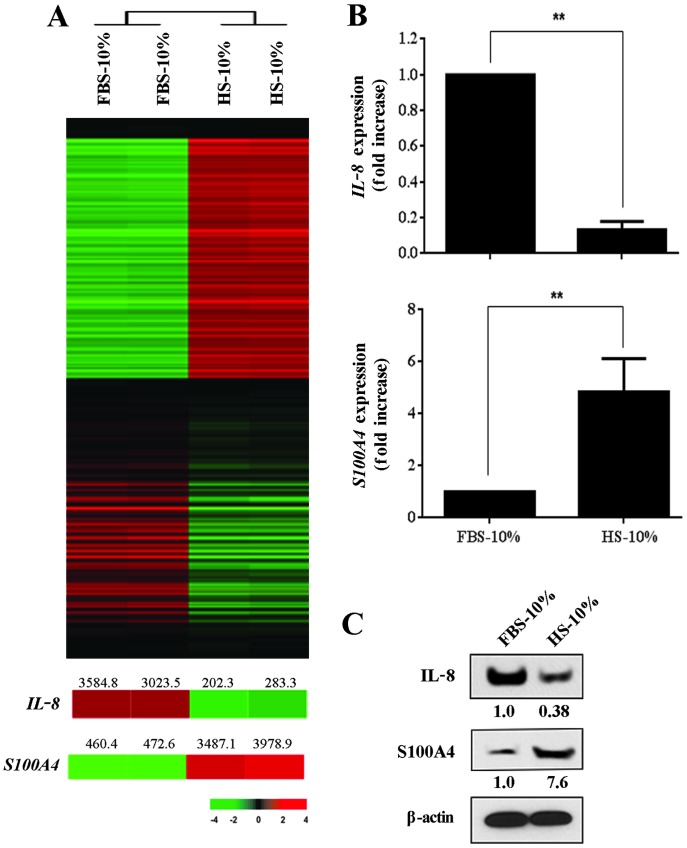
Differential gene expression between MDA-MB-231 breast cancer cells cultured in minimum essential medium (MEM) supplemented with 10% fetal bovine serum (FBS) and cells cultured in MEM containing 10% horse serum (HS). (A) Hierarchical clustering analysis of cells maintained in 10% FBS and 10% HS. Genes with an expression level exceeding a 2-fold difference relative to the median value were selected for hierarchical clustering analysis (n=2,981 genes). (B) Real-time reverse transcription polymerase chain reaction (RT-PCR) analysis of interleukin-8 (*IL-8*) and *S100A4*. (C) Western blot analysis of IL-8 and S100A4. Values shown are means ± standard deviation (SD) from three independent experiments. ^**^P<0.01.

Virtually any type of animal is capable of serving as a donor for serum, but some animal sera are used more often than others. Fetal bovine serum (FBS) and horse serum (HS) are among the more common types of sera used for culturing mammalian cells and we evaluated the effects of these sera on MDA-MB-231 cancer cell gene expression. In this series of experiments, we maintained the density of cell cultures at the time of analysis constant at 90%. While altering the source of the sera used in cell culture significantly affected cancer cell gene expression, the overall effect was less than that observed when we modified the concentration of FBS in the culture media or when we analyzed cells grown at different cell densities. We recorded a total of 422 differentially expressed genes in a comparison between MDA-MB-231 cancer cells grown in MEM containing 10% FBS and those grown in MEM containing 10% HS. A total of 235 and 187 genes were significantly upregulated and down-regulated, respectively, in cancer cells in the 10% FBS cell group (Fig. 3A). *IL-8* and *S100A4* were among the differently modulated genes and expression levels were confirmed using RT-PCR (Fig. 3B) and western blot analysis (Fig. 3C). IL-8 protein expression in MDA-MB-231 cancer cells grown in media supplemented with 10% FBS was ~3-fold higher than of cells maintained in 10% HS. In contrast, S100A4 protein expression was negligible in MEM containing FBS, but was markedly upregulated in media containing HS.

### Cell culture media formulations exert the most profound effect on cancer cell gene expression

**Figure 4:**
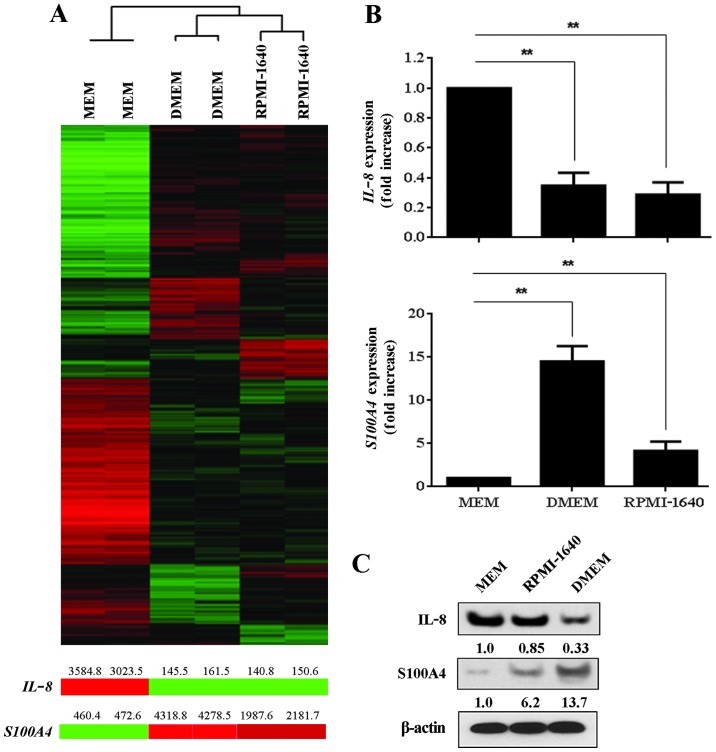
Differential gene expression between MDA-MB-231 breast cancer cells maintained in different media formulations. (A) Hierarchical clustering analysis of MDA-MB-231 breast cancer cells cultured in minimum essential medium (MEM), Dulbecco’s modified Eagle’s medium (DMEM), and Roswell Park Memorial Institute (RPMI)-1640 medium that were all supplemented with 10% fetal bovine serum (FBS). (B) Real-time reverse transcription polymerase chain reaction (RT-PCR) analysis of interleukin-8 (*IL-8*) and *S100A4*. (C) Protein levels of IL-8 and S100A4 were measured by western blot analysis. Values shown are means ± standard deviation (SD) from three independent experiments. ^**^P<0.01.

To determine how different media formulations affect cancer cell gene expression, we cultured MDA-MB-231 breast cancer cells in MEM, DMEM, or RPMI-1640 media, all containing 10% FBS, and when the cultures reached 90% confluence, we extracted total RNA from the cells and applied it to beadchip microarrays for analysis. A total of 8,925 genes were differentially expressed when we compared microarray results from MDA-MB-231 cancer cells grown in MEM with those cells grown in DMEM or RPMI-1640 (Fig. 4A). Specifically, 1,409 genes were highly expressed in MEM as compared to DMEM and RPMI-1640; 840 genes were highly expressed in DMEM as compared to MEM and RPMI-1640; and 1,662 genes were highly expressed in RPMI-1640 as compared to MEM and DMEM. The microarray analysis predicted IL-8 expression levels would be greatest in MDA-MB-231 cells that were grown in MEM and least in cells that were maintained in DMEM and RPMI-1640 and this finding was confirmed by RT-PCR (Fig. 4B). *S100A4* gene expression was significantly greater in MDA-MB-231 cells grown in DMEM and RPMI-1640 in comparison to cells grown in MEM and this was also validated by RT-PCR (Fig. 4B). Indeed, *S100A4* gene expression levels were 15- and 5-fold greater in cells grown in DMEM and RPMI-1640, respectively, as compared to cells grown in MEM. Western blot analysis of IL-8 and S100A4 paralleled the gene expression results (Fig. 4C).

### Cell culture media formulations modulate expression of genes associated with EMT

**Figure 5:**
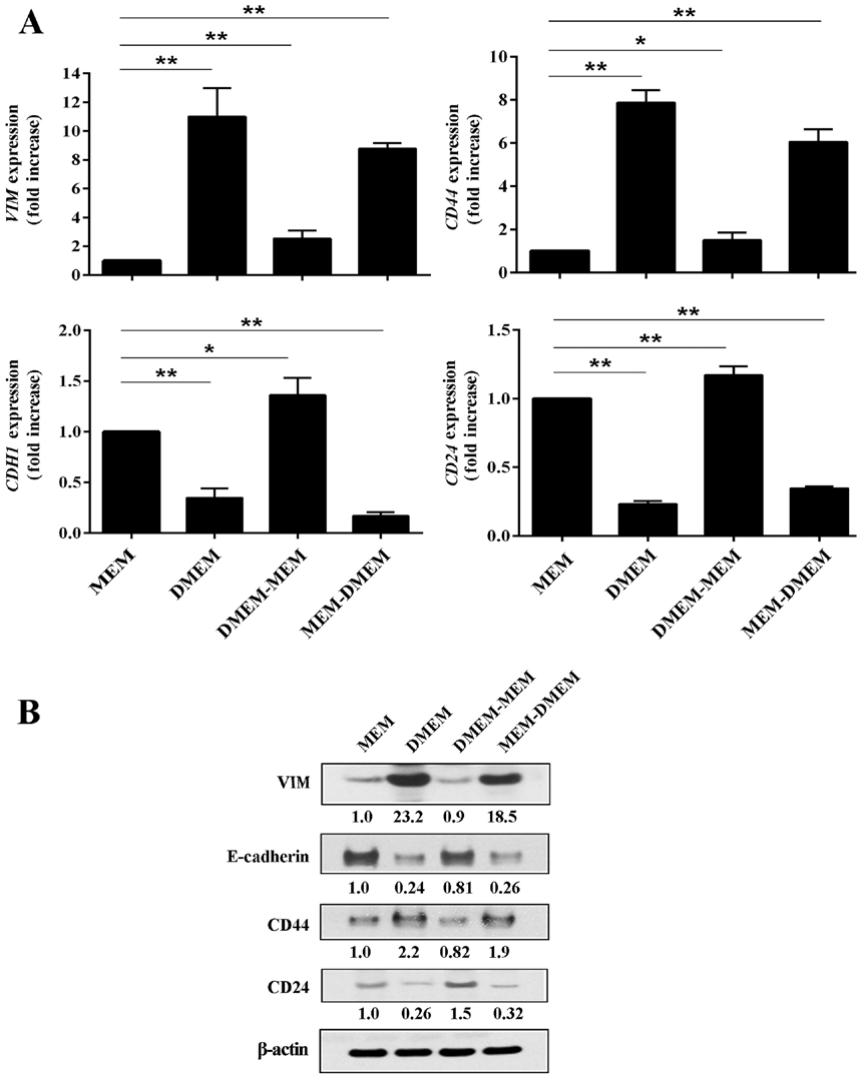
Expression of epithelial-mesenchymal transition (EMT) markers in MDA-MB-231 breast cancer cells cultured in minimum essential medium (MEM) and Dulbecco’s modified Eagle’s medium (DMEM) formulations. (A) MDA-MB-231 breast cancer cells were cultured in MEM or DMEM containing 10% fetal bovine serum (FBS) for 48 h. Real-time reverse transcription polymerase chain reaction (RT-PCR) analysis of vimentin (*VIM)*, *CDH1*, *CD44*, and *CD24* gene expression. To determine if changes in gene expression were reversible, the culture media was changed from MEM to DMEM (or from DMEM to MEM) and the cells were analyzed 48 h later. Fold-increase refers to the ratio of mRNA levels relative to that of cells cultured in MEM. (B) Western blot analysis comparing protein expression of VIM, E-cadherin, CD44, and CD24. ^*^P<0.05, ^**^P<0.01.

Data mining on the expression array sets suggested that genes frequently associated with epithelial-mesenchymal transition (EMT) were differentially modulated by the various media formulations. MDA-MB-231 cancer cells that were grown in MEM had a tendency to express significantly higher expression levels of epithelial cell markers E-cadherin (*CDH1*) and *CD24*, whereas the same cells grown in DMEM expressed higher levels of mesenchymal markers *VIM* and *CD44*. We confirmed the gene expression array using RT-PCR analysis. Gene expression levels of *VIM* and *CD44* in MDA-MB-231 cancer cells were dramatically suppressed when the cells were grown in MEM in comparison to cells grown in DMEM (Fig. 5A, upper panel). *VIM* and *CD44* expression levels were at least 8-fold higher in cells that were maintained in DMEM. However, expression levels of these two genes could be easily modulated by simply switching the cells to a different media formulation for a period of 48 h. When DMEM was replaced by MEM, expression levels of *CD44* and *VIM* dropped dramatically; when MEM was replaced by DMEM expression, levels of *CD44* and *VIM* increased.

The gene expression patterns of *CDH1* and *CD24* were diametrically opposed to that of *CD44* and *VIM*. That is, *CDH1* and *CD24* gene expression were elevated in MDA-MB-231 cancer cells when they were grown in MEM and both were suppressed when cells were maintained in DMEM (Fig. 5A, lower panel). Similar to our analysis of *CD44* and *VIM*, gene expression of *CDH1* and *CD24* was plastic and influenced by the *in vitro* microenvironment. When the MDA-MB-231 cancer cell media was changed from MEM to DMEM for 48 h, expression levels of *CDH1* and *CD24* were significantly reduced. All of the gene expression results were validated at the protein level by western blot analysis (Fig. 5B).

## Discussion

Cancer cell lines play an invaluable role in the drug discovery process (16) and in identifying molecular mechanisms of therapeutic resistance (7). Because tumor cell lines consist of pure populations of cancer cells, they are frequently more advantageous for study as compared to tumor tissue (8). For example, cancer cell lines provide a source of high quality DNA, RNA, and proteins that may facilitate testing and data interpretation (8). However, the misidentification of cell lines and their cross-contamination have led to a number of misleading and erroneous publications in the scientific literature (17,18). Recently, several granting agencies and scientific journals have sought to handle this problem by requiring investigators to provide cell line authentication for human cancer cells using short tandem repeat profiling (18). In the present report, we provide convincing evidence that the replication of results on cultured cancer cells also requires rigid adherence to the cell culture formulation used to maintain cells and reporting of the cell density reached at the time of analysis.

One of the more striking observations of our study was that in our whole-genome microarray analysis of MDA-MB-231 breast cancer cells, approximately one-quarter (25.6%) of all genes were differentially expressed when we examined cells that were grown in different media formulations. While we had predicted that several genes would be differentially modulated by the various media preparations, we did not anticipate the extent of differential gene expression observed in our study. Genes associated with the EMT program were upregulated or downregulated simply by switching the cells to a different media formulation (different concentration of glucose) for a period of 48 h. EMT plays a critical role during development and is also observed in the process of tissue repair (19). Invading and metastasizing carcinoma cells revive the EMT program by upregulating mesenchymal proteins and by suppressing expression of epithelial proteins (20). EMT has been the focus of much recent investigation because cancer cells undergoing EMT have been shown to obtain stem cell-like properties and become resistant to anticancer agents (21). Our results demonstrate that EMT gene and protein expression is remarkably plastic in cultured cancer cells and that EMT is highly influenced by the tissue culture microenvironment. It is tempting to speculate that the elevated glucose concentration present in DMEM and RPMI-1640 relative to that found in MEM was responsible for the induction of the EMT program in MDA-MB-231 breast cancer cells. Evidence to support this contention comes from studies of renal tubular cells (22) and peritoneal mesothelial cells (23), which activate the EMT program in response to stimulation with high concentrations of glucose. Moreover, a recent study demonstrated that the small fraction of cancer cells with stem-like properties that exists in a tumor could be dramatically increased in the cell culture environment by culturing the cells in high concentrations of glucose (24). These investigations lend credibility to the experimental approach used in our study and suggest that additional study in this area is warranted.

Two genes that have been linked to breast cancer progression, *IL-8* and *S100A4*, were differentially modulated by every cell culture modification that we examined. S100A4 is a small calcium-binding protein that has been shown to promote migration, invasion, and anchorage-independent growth of breast cancer cells (25). In the present report, *S100A4* was upregulated in MDA-MB-231 cancer cells under conditions of low cell density, minimal FBS supplementation, HS, and when cells are grown in a DMEM formulation. Alternatively, *S100A4* expression was negligible when cells were maintained in media supplemented with 10% FBS and when cells achieved a confluent state. IL-8 is a human chemokine that is produced by a variety of different cell types. In healthy tissues, IL-8 is minimally expressed, but can be rapidly induced 100-fold in response to pro-inflammatory cytokines, such as tumor necrosis factor, IL-1, bacterial or viral products, and cellular stress (26). IL-8 contributes to the progression of several types of tumors by mediating cancer cell migration and stimulating tumor neovascularization (27). More recent studies have shown that IL-8 signaling may function as a key factor in the regulation of breast cancer stem cell activity (28). In our study, IL-8 expression was enriched in confluent MDA-MB-231 breast cancer cells that were maintained in MEM containing 10% FBS.

Collectively, our results show that the gene expression patterns of cancer cells can vary significantly according to the conditions under which they are cultured. While the introduction of measures that ensure the identification of cancer cell lines used in research investigations will undoubtedly improve the reproducibility of translational oncology research, accurate documentation of cell culture conditions is essential for replicating results from *in vitro* studies of cancer cells.
